# Shaping Clinical Policy for Salvage Radiotherapy After Radical Prostatectomy in Prostate Cancer: Bridging the Gap Between Clinical Trials and Daily Practice

**DOI:** 10.1002/cam4.70362

**Published:** 2024-11-01

**Authors:** Piero Bettoli, Evangelina Röling, Moises Russo, María Fernanda Sánchez, Inti Paredes, Jorge Sapunar

**Affiliations:** ^1^ Department of Radiation Oncology Arturo López Pérez Foundation Cancer Centre Santiago Chile; ^2^ Department of Cancer Research Arturo López Pérez Foundation Cancer Centre Santiago Chile; ^3^ Department of Digital Health and Medical Informatics Arturo López Pérez Foundation Cancer Centre Santiago Chile

**Keywords:** clinical trials, prostate cancer, radiotherapy, surgery

## Abstract

**Purpose and Objective:**

Salvage radiotherapy (sRT) can have similar outcomes to adjuvant radiotherapy (aRT) if administered at the earliest evidence of biochemical recurrence. RADICALS‐RT was the first trial to support this hypothesis and a policy of observation after radical prostatectomy (RP) with early sRT has become the new standard of care since then. This study assessed the impact of RADICALS‐RT in the clinical practice regarding the timing of sRT for prostate cancer initially treated with RP.

**Methods:**

Data from 297 patients who underwent sRT after radical RP were retrospectively collected. Two groups were created and analyzed on the basis of the date of RADICALS‐RT presentation at ESMO. After these results were released in October 2021, our institutional postoperative radiotherapy policy was revisited, and a third group was created and analyzed separately.

**Results:**

Median PSA for Groups 1, 2, and 3 were 0.33, 0.27, and 0.2, respectively. Less than one‐third of patients in Groups 1 and 2 had a postoperative PSA of 0.2 ng/mL or less at the time of sRT. Group 3 showed statistically significant differences in median PSA at the time of sRT compared with Groups 1 and 2.

**Conclusions:**

RADICALS‐RT demonstrated a significant impact on clinical practice only after being complemented with real local evidence.

## Graphical abstract

In this report, we looked at the impact of RADICALS‐RT regarding the timing of sRT for prostate cancer initially treated with RP. We found that RADICALS‐RT has influenced clinical practice and led to more timely administration of sRT only after being complemented with real local evidence. We conclude that a combination of randomized controlled trials (RCTs) and real‐world data (RWD) can provide a more comprehensive understanding of the effectiveness of different treatment strategies and help refine clinical practice guidelines.

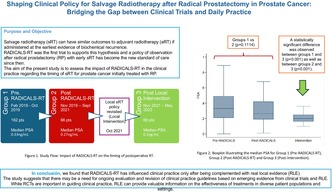

Prostate cancer is a significant health concern globally, with an estimated 1.4 million new cases in 2020 alone [1]. Radical prostatectomy (RP) is a common treatment option for localized prostate cancer, but it is associated with a risk of biochemical recurrence (BCR) [2].

Adjuvant radiotherapy (aRT) has been investigated in several randomized controlled trials (RCTs) and has been shown to significantly improve the long‐term outcomes of patients with prostate cancer who have undergone RT. However, aRT has been associated with increased toxicity, including gastrointestinal and genitourinary side effects, and its efficacy and potential benefits have been questioned [3, 4, 5].

Recent studies have shown that salvage radiotherapy (sRT) has similar outcomes to aRT in terms of overall survival, distant metastasis‐free survival, and prostate cancer‐specific survival with less urinary and bowel morbidity [6]. RADICALS‐RT, a large multicenter RCT, was the first trial to provide evidence that sRT administered at the earliest evidence of BCR (median PSA 0.2 ng/mL) can have similar outcomes to aRT [7]. As a result, a policy of observation after RP with early sRT has become the new standard of care.

The impact of RADICALS‐RT on the clinical practice regarding the timing of sRT for prostate cancer initially treated with RP remains unclear. Thus, the aim of the present study was to assess this impact and contribute to the understanding of the optimal timing of sRT for patients with prostate cancer.

The present investigation employed an observational and analytical methodology, utilizing a longitudinal design and a retrospective cohort of patients who received sRT after RP at a single institution over 7 years, from February 2016 to September 2022. Institutional Ethics Committee approval was obtained for retrospective review of data. Patient demographics, disease‐related clinical information, and PSA level before sRT were collected. Patients with non‐metastatic prostate cancer were eligible if they had undergone RP, received sRT given later to those who develop a rising PSA, and had at least one specified risk factor (pathological T‐stage: 3 or 4, Gleason score: 7–10, positive margins, or preoperative PSA of 10 ng/mL or more).

Patients were divided into two groups on the basis of the date of RADICALS‐RT presentation at ESMO (November 2019). Two groups were created and analyzed separately: pre‐RADICALS‐RT group from February 2016 to October 2019 (Group 1), and post‐RADICALS‐RT group from November 2019 to September 2021 (Group 2). After these results were released in October 2021, our genitourinary institutional group revisited our postoperative radiation therapy policy. A third group (Group 3) was created and analyzed separately from the previous ones after this institutional intervention (from November 2021 to May 2023).

The study included a total of 297 patients who were recruited to participate. The median age at the time of diagnosis was 67 years. Median PSA at diagnosis was 8 ng/mL. Pathological T‐stage distribution showed that 41.5% of patients were classified as T2, 27.7% as T3a, 30% as T3b, and 0.9% as T4. The distribution of Gleason scores was as follows: 7.5% had a Gleason score of 6, 67.7% had a Gleason score of 7%, and 24.8% had a Gleason score of 8 or greater. Positive margins were found in 46% of the patients. The median PSA level for the entire cohort at the time of sRT was 0.27 ng/mL (Table 1).

**TABLE 1:** Baseline characteristics.

	Group 1 (*n* = 162)	Group 2 (*n* = 67)	Group 3 (*n* = 69)	All (*n* = 298)
Age, years (median)	67.6	66.3	65.3	66.7
PSA at diagnosis, ng/mL (median)	8.45	8	7.25	7.6
Gleason score				
< 7	14 (8.6%)	3 (4.5%)	6 (8.7%)	23 (7.8%)
3 + 4	60 (37%)	27 (40.3%)	27 (39.1%)	114 (38.3%)
4 + 3	42 (26%)	23 (34.3%)	24 (34.8%)	89 (29.8%)
≥ 8	45 (27.8%)	11 (16.4%)	10 (14.5%)	66 (22.1%)
Unknown	1 (0.6%)	3 (4.5%)	2 (2.9%)	6 (2%)
Pathological T‐stage				
2	66 (40.7%)	28 (41.8%)	34 (49.2%)	128 (43%)
3a	43 (26.5%)	20 (29.9%)	11 (16%)	74 (24.8%)
3b	50 (30.9%)	15 (22.5%)	20 (29%)	85 (28.6%)
4	0 (0%)	2 (2.9%)	2 (2.9%)	4 (1.3%)
Unknown	3 (1.9%)	2 (2.9%)	2 (2.9%)	7 (2.3%)
Positive margins				
Present	77 (47.5%)	31 (46.3%)	28 (40.5%)	136 (45.6%)
Absent	85 (52.5%)	28 (41.8%)	34 (49.3%)	147 (49.3%)
Unknown	0 (0%)	8 (11.9%)	7 (10.2%)	15 (5.1%)
Lymph node involvement				
Node negative	112 (69.2%)	47 (70.2%)	48 (69.6%)	207 (69.5%)
Node positive	25 (15.4%)	6 (8.9%)	7 (10.2%)	38 (12.7%)
No dissection	25 (15.4%)	14 (20.9%)	14 (20.2%)	53 (17.8%)

One hundred and sixty‐two patients were included in Group 1, 66 patients in Group 2, and 69 patients in Group 3. Median PSA for Groups 1, 2, and 3 were 0.33, 0.27, and 0.2, respectively. Less than one‐third of patients in Groups 1 and 2 (24% and 29%, respectively) had a postoperative PSA of 0.2 ng/mL or less at the time of sRT. When comparing the three groups, the Kruskal–Wallis test revealed a significant difference (*p* < 0.001) between them. Subsequent Dunn tests indicated no statistically significant difference between Groups 1 and 2 (*p* = 0.1114). However, a statistically significant difference was observed between Groups 1 and 3 (*p* < 0.001) as well as between Groups 2 and 3 (*p* < 0.001) (Figure 1).

**FIGURE 1:**
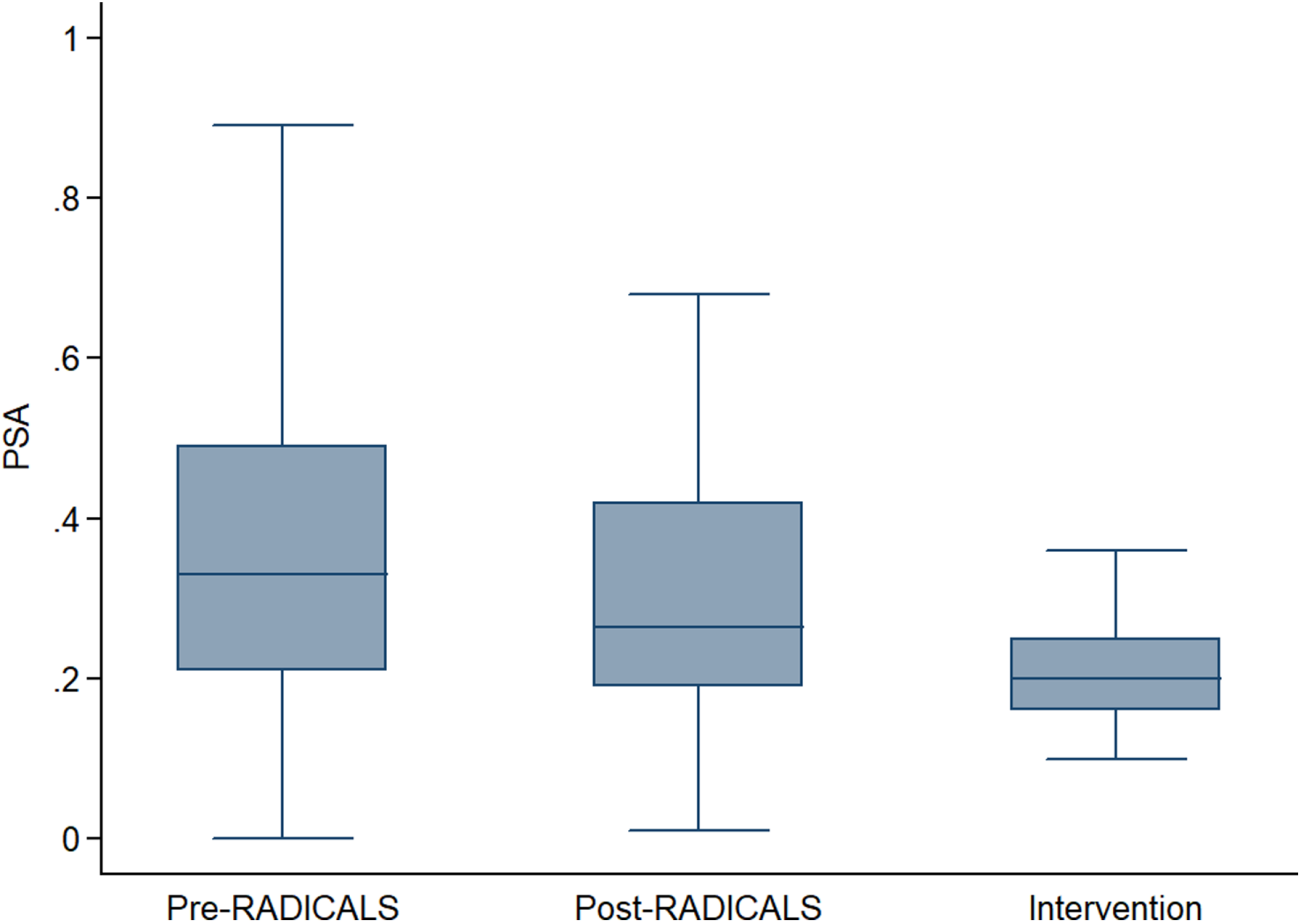
Boxplot illustrating the median PSA for Group 1 (Pre‐RADICALS‐RT), Group 2 (Post‐RADICALS‐RT), and Group 3 (Post‐Intervention).

Despite the growing evidence supporting the use of sRT, the optimal timing for initiating sRT remains a controversial issue and is often based on individual patient and disease characteristics.

It is well known that the timing of sRT after a RP matters [8]. The American Society of Radiation Oncology (ASTRO), The American Urological Association (AUA), and The European Urological Association (EUA) have addressed this issue, and they all advise initiating sRT when the PSA level reaches 0.2 ng/mL or higher following RP [9, 10]. Nonetheless, recent studies suggest that commencing sRT at a lower PSA threshold may produce better results [11]. In the RADICALS‐RT study, the earliest evidence of BCR was defined as the occurrence of either two consecutive PSA level increases, with a PSA exceeding 0.1 ng/mL, or three consecutive PSA level increases. The median PSA for the entire cohort at the time of recurrence was 0.2 ng/mL [7].

The present study found that a significant proportion of patients in the earlier two groups did not receive sRT at the earliest evidence of BCR, as recommended by the trial [7]. However, after an institutional intervention following the release of these results, the subsequent patients recruited (Group 3) showed a significantly lower median PSA at the time of sRT. These results could be explained due to lack of knowledge of the RADICALS‐RT trial, reluctance to change established clinical practice, or difficulties in timely referral and access to sRT. The results of the third group, which received sRT after an institutional intervention, suggest that the RCT has influenced clinical practice and led to more timely administration of sRT but only after being complemented with real local evidence.

RCTs and real‐world data (RWD) are two different methods of studying the safety and effectiveness of medical interventions. Although RCTs provide strong evidence of the efficacy of interventions, RWD provides insights into the effectiveness of interventions in a broader population and in real‐world conditions. Both approaches have their strengths and limitations and can complement each other in providing a comprehensive understanding of the safety and efficacy of medical interventions [12].

The findings of our study highlight the importance of keeping up‐to‐date with the latest clinical trial evidence and the need for integrate RWD to ensure that patients receive the best possible care [12]. Specifically, for patients who undergo RP, clinicians should be careful in adopting a sRT approach if conditions are not appropriate, and they should reconsider an aRT strategy in this context.

This study has several limitations, including its retrospective design and the single‐center source of data. Additionally, the study did not evaluate the impact of RADICALS‐RT on other aspects of clinical practice, such as referral patterns or patient education. Future studies could assess these factors to provide a more comprehensive understanding of the trial's influence on clinical practice.

In summary, the timing of sRT after RP is a critical factor in achieving successful outcomes in patients with prostate cancer. We found that RADICALS‐RT has influenced clinical practice and led to more timely administration of sRT only after being complemented with real local evidence. The study suggests that there may be a need for ongoing evaluation and revision of clinical practice guidelines based on emerging evidence from clinical trials and RWD. Although RCTs are important in guiding clinical practice, RWD can provide valuable information on the effectiveness of treatments in diverse patient populations and settings.

## Author Contributions

**Piero Bettoli:** conceptualization (lead), investigation (lead), methodology (lead), project administration (lead), supervision (lead), visualization (lead), writing – original draft (lead). **Evangelina Röling:** methodology (supporting), writing – review and editing (lead). **Moises Russo:** formal analysis (lead), writing – original draft (supporting). **María Fernanda Sánchez:** investigation (supporting), writing – review and editing (supporting). **Inti Paredes:** resources (supporting), writing – review and editing (supporting). **Jorge Sapunar:** writing – review and editing (supporting).

## Conflicts of Interest

The authors declare no conflicts of interest.

## Data Availability

The data that support the findings of this study are available on request from the corresponding author. The data are not publicly available due to privacy or ethical restrictions.
